# Delivering Patient-Centered Care in a Fragile State: Using Patient-Pathway Analysis to Understand Tuberculosis-Related Care Seeking in Pakistan

**DOI:** 10.1093/infdis/jix380

**Published:** 2017-11-06

**Authors:** Razia Fatima, Mahboob Ul Haq, Aashifa Yaqoob, Nasir Mahmood, Khawaja Laeeq Ahmad, Mike Osberg, Julia Makayova, Aaron Hymoff, Christy Hanson

**Affiliations:** 1 National Tuberculosis Control Program, Islamabad, Pakistan; 2 Linksbridge; 3 Bill and Melinda Gates Foundation, Seattle, Washington; 4 Macalester College, St Paul, Minnesota

**Keywords:** Tuberculosis, patient-pathway analysis, care seeking, diagnostic, case notification, private, public

## Abstract

**Background:**

Pakistan has the sixth largest population in the world and boasts the fifth greatest burden of tuberculosis. The Government of Pakistan has set the ambitious goal of zero deaths due to tuberculosis and universal access to tuberculosis care by 2020. Successfully reaching these goals is dependent on the country’s capacity to diagnose and successfully treat an estimated 200000 unnotified or missing patients with tuberculosis.

**Methods:**

A patient-pathway analysis (PPA) was conducted at the national level, as well as for each of the 4 provinces, to assess the alignment between patient care seeking and the availability of tuberculosis diagnostic and treatment services.

**Results:**

Almost 90% of patients initiated care in the private sector, which accounts for only 15% of facilities with the capacity for tuberculosis diagnosis and treatment. Across the country, nearly 50% of tuberculosis microscopy laboratories were located in public-sector–basic health units and regional health centers. However, very few patients initiated care in these facilities. Overall, tuberculosis case detection was high given the low likelihood of patients reaching facilities with the capacity for tuberculosis service delivery during their first visit.

**Discussion:**

Improving the engagement of the informal sector and lower-level clinicians will improve the efficiency and timeliness of tuberculosis diagnosis for patients in Pakistan. Concurrently, the apparent strength of the referral networks connecting community-level workers and private clinicians to the public sector for tuberculosis diagnosis and treatment suggests that strengthening the capacity of the public sector could be valuable.

Pakistan has the sixth largest population in the world [1]. Access to health services remains inequitable; 62% of the population lives in rural areas with poor access to healthcare, and continued socioeconomic and political volatility limits access for some Pakistanis [2]. While the country has slowly stabilized, Pakistan was still ranked the 14th most fragile state in 2016 [3]. However, Pakistan has made significant gains in poverty reduction, with a reduction from 64% of the population in 2002 to >30% in 2015 [4].

Tuberculosis is the ninth leading cause of death in Pakistan [5]. Pakistan ranks fifth globally in terms of the absolute number of tuberculosis cases and is also estimated to have the fourth highest prevalence of multidrug-resistant tuberculosis in the world [6]. In 2015, the estimated number of new drug-susceptible tuberculosis cases in Pakistan was 510000, with 63% of those cases notified to the National Tuberculosis Program (NTP). In addition, Pakistan had an estimated 14000 cases of drug-resistant tuberculosis among notified pulmonary tuberculosis cases, and the World Health Organization (WHO) estimated that 4.2% of all new tuberculosis cases and 16% of retreatment cases were multidrug resistant [7]. Despite these distinct challenges, the treatment success rate for drug-susceptible tuberculosis was 91% in 2015 [7].

The End TB Strategy promoted by the WHO emphasizes the importance of patient-centered care [8]. Identifying and curing missing patients with tuberculosis requires a robust understanding of how patients interact with the healthcare system and how the provision of tuberculosis services can be designed to meet patients where they are.

There were approximately 180000 undetected patients with tuberculosis in Pakistan in 2015, which accounted for about 5% of missing cases globally [9]. Ensuring successful diagnosis and treatment of all missing cases is a stated priority for the Government of Pakistan. The ambitious National TB Control Strategic Plan, Vision 2020, outlines the objectives of universal access to tuberculosis care and zero tuberculosis deaths by 2020 [6].

Pakistan maintains a decentralized governance structure composed of 5 provinces and 3 regions. The public health system in Pakistan is similarly decentralized. Public primary care is delivered by basic health facilities, such as rural health units, maternal health centers, child health centers, and dispensaries [10]. These are supported by district-level secondary care facilities, provincial-level tertiary care facilities, and central-level referral hospitals. Lady health and community health workers (LHWs) provide mostly maternal, newborn, and child healthcare. LHWs have been enlisted to help identify individuals presumed to have tuberculosis, to refer them to health centers for screening or diagnosis, and to support tuberculosis treatment [11, 12]. They have wide coverage throughout the country and provide a direct linkage between communities and public health centers.

The private sector is large and unregulated, comprising qualified and unqualified service providers that deliver general curative services to about 75% of Pakistan’s population [10]. There are almost 10000 private hospitals and diagnostic laboratories in Pakistan, representing approximately 83% of all facilities in the nation [10]. Despite the large share of the health sector that they occupy, <1% of private providers report tuberculosis cases to the NTP [6, 9].

## METHODS

Patient-pathway analyses (PPAs) were completed for Pakistan at the national level and for 4 of 5 provinces—Balochistan, Khyber Pakhtunkhwa, Sindh, and Punjab. Each PPA included 7 data points and focused on the alignment of patients’ care initiation patterns for general illness with the location of tuberculosis diagnostic tools (ie, microscopy). The data sources for these metrics are listed in Table 1, and descriptions of the necessary calculations are included below. A more detailed description of the PPA method is provided elsewhere in this supplement by Hanson et al [13], and further background on each data source is provided in the Supplementary Materials.

**Table 1.** Data Sources for the Patient-Pathway Analysis (PPA)

PPA Component	Data Source(s)
Care seeking for general illness	*National Health Accounts 2011–2012* [14]
Tuberculosis microscopy location	Pakistan National Tuberculosis Program Laboratory database
Tuberculosis treatment services location	Pakistan National Tuberculosis Program Laboratory database, internal Pakistan National Tuberculosis Program list of general practitioners with tuberculosis treatment services
No. of health facilities	*National Health Accounts 2009–2010* (private-sector facilities) [15], *National Health Accounts 2011–2012* (public-sector facilities) [14]
Case detection rate	World Health Organization *Global Tuberculosis Report 2016* [7]

Each of the data sources used a different naming convention for health facilities. To allow for comparison across data sources, common categories were created to designate individual facilities as being either public, private (formal), or private (informal) and to classify them as belonging to one of the 3 levels in the health system. Level 0 (L0) refers to the most basic care level, which is usually community based. Level 0 services include basic triage, provision of health information, and essential prevention activities and care. Services are commonly provided as an extension of facility-based care and are provided by volunteers or paramedical staff with limited formal training. No laboratory testing is available, but L0 staff may serve as treatment supporters for patients with tuberculosis. Examples include hakim, herbalist, and homeopath clinics; pharmacies; and shops (private). Level 1 (L1) facilities provide primary healthcare. Nurses, midwives, or private physicians commonly provide L1 services, generally on an outpatient basis. Some basic diagnostic services and essential medicines may be available. Examples include basic health units and rural health centers (public) and private physician clinics (private). Level 2 (L2) facilities provide primary healthcare, as well as more-advanced care. L2 facilities commonly have more-extensive diagnostic and treatment options and can provide both outpatient and inpatient care. Examples are public hospitals (public) and nongovernmental organizations, private hospitals, and laboratories (private). Other than the list of tuberculosis facilities provided by the NTP, the data sources did not separate hospitals by L2 and L3 hospitals. Thus, all hospitals were categorized as L2. Table 2 shows the detailed mapping of health facilities from each data source.

**Table 2.** Health Facility Categorization

Data Source, Health Facility Type	Health Facility Sector	Health Facility Level
*National Health Accounts 2011–2012* [14]		
Care seeking		
Other	Other	Other
Homeopathic, hakim, herbalist clinics	Informal private	0
Pharmacy, shops	Informal private	0
Laboratory	Private	2
Private hospital	Private	2
Private physician clinic	Private	1
Government hospital, *tehsil* headquarters hospital, district headquarters hospital, tertiary-care hospital, teaching hospital	Public	2
Dispensary, maternal and child health center, basic health unit, rural health center	Public	1
Private sector		
Hakim, herbalist clinics	Informal private	0
Homeopathic clinic	Informal private	0
Traditional birth attendant	Informal private	0
Hospital	Private	2
Laboratory	Private	2
Dental clinic	Private	1
Individually run solo clinic (allopathic clinics)	Private	1
Other	Private	1
Outpatient care center	Private	1
Hospital	Public	2
Dispensary	Public	1
Maternity and child welfare center	Public	1
Public sector		
Hospital	Public	2
Basic health unit	Public	1
Dispensary	Public	1
Maternity and child welfare center	Public	1
Rural health center	Public	1
Tuberculosis clinic	Public	1
National Tuberculosis Program database of tuberculosis facilities		
District headquarters hospital	Private	2
Hospital	Private	2
Tertiary-care hospital	Private	2
*Tehsil* headquarters hospital	Private	2
Basic health unit	Private	1
Basic management unit	Private	1
Public-private mix	Private	1
Basic health unit	Private	1
Rural health center	Private	1
Jail	Private	Other
Civil hospital	Public	2
District headquarters hospital	Public	2
Hospital	Public	2
Hospital^a^	Public	2
Hospital (unknown type)	Public	2
Major hospital	Public	2
Tertiary-care hospital	Public	2
Teaching hospital	Public	2
*Tehsil* headquarters hospitals	Public	2
Basic health unit	Public	1
Basic management unit	Public	1
Dispensary	Public	1
Public-private mix	Public	1
Rural health center	Public	1
Tuberculosis center	Public	1
Jail	Public	Other

^a^See notes.

**Figure 1.**
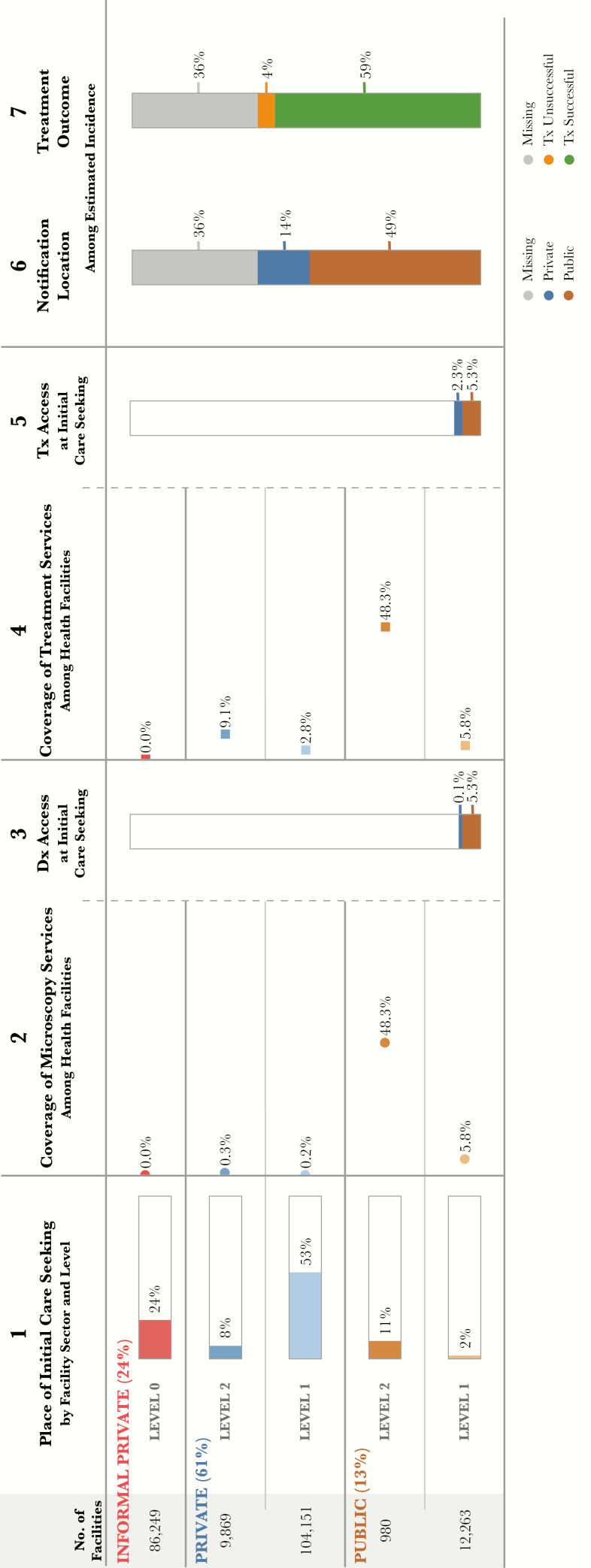
Patient-pathway visual at the national level. The patient pathway describes the care-seeking patterns of patients and how those patients might intersect with tuberculosis services. Column 1 first shows the sectors and levels of the health system (sectors and levels where no data were available are not included in the pathway). The percentage next to each sector title shows the share of patients who initiate care seeking in this sector [14]. Next are the reported numbers of health facilities at each sector and level [14, 15]. The final part of column 1 shows the percentage of patients at each sector and level who visited a health facility during the 4 weeks before a national health accounts out-of-pocket expenditure survey in 2011–2012 [14]. Column 2 shows the percentage of health facilities that have microscopy across each sector and level of the health system. Coverage in column 2 was calculated using the number of health facilities in column 1 as the denominator and, as the numerator, a list of health facilities with tuberculosis services (microscopy and treatment) provided by the national tuberculosis program. This list included both public and private facilities [16]. Column 3 shows the estimated percentage of patients likely to access a facility with tuberculosis diagnosis available on their first visit to a healthcare facility. This column was calculated by multiplying the share of care seeking at each sector/level of the health system by the coverage of microscopy at each respective sector/level and summing the total. Columns 3 and 5 separate public and private sectors on the basis of each sector’s contribution to tuberculosis services access at initial care seeking. Column 4 shows the percentage of health facilities that have tuberculosis treatment available at each sector and level of the health system. Coverage of treatment services was calculated using the same data from column 2 [16]. In addition, the National Tuberculosis Program maintains an additional list of private facilities that are engaged to provide tuberculosis treatment services as part of public-private mix programs. This list was included as part of the treatment coverage calculations for private-sector facilities. Column 5 shows the estimated percentage of patients accessing a facility with tuberculosis treatment available on their initial visit to a healthcare facility. This column was calculated by multiplying the share of care seeking at each sector/level of the health system by the coverage of treatment at each respective sector/level and summing the total. Column 6 shows which sector provided case notification, and values are calculated as a share of the overall estimated incidence in 2015 [7]. Column 7 shows the treatment outcome of notified cases among the overall estimated incidence for 2015 [7]. Columns may not sum to 100%, owing to rounding. For more details on the data sources used in the pathway, see the Supplementary Materials.

Using data from 2 National Health Accounts (NHA) surveys, the reported number of health facilities is included at the beginning of the patient pathway visual (column 1, Figure 1) [14, 15]. To estimate the location of care initiation, the PPA used data from the 2011–2012 National Health Accounts report [14]. The survey asked participants where they initiated care for general illness. The data were available for 4 provinces and at the national level. Respondents were given several options for different types of public and private health facilities, which were grouped into the categories described above for use in the PPA. These estimates were then used in the PPA as a proxy for where individuals presumed to have tuberculosis initiate care (Figure 1).

Three data sources were combined to calculate the microscopy coverage at each health facility level. The first, the 2009–2010 NHA, provided data on the number of health facilities in the private sector [15]. The 2009–2010 version was used because the more recent 2011–2012 NHA, which was used to estimate care-seeking patterns, did not include an updated on the number of private-sector facilities. The second source, the 2011–2012 NHA, provided data on the number of health facilities in the public sector [14]. These data sources provided a breakdown of different types of health facilities by sector, which were then categorized using the standard definitions described above. Both sources provided the denominator values for the coverage metric.

The NTP maintains a list of all tuberculosis microscopy facilities throughout the country. This list was used as the third data source, to develop the numerator of the microscopy coverage metric. Each microscopy facility was categorized by type, level, and sector [16]. To estimate microscopy coverage, the number of microscopy facilities was divided by the total number of facilities in each province, sector, and level. These quotients were used for column 2 of the PPA (Figure 1).

The third column of the PPA estimates the likelihood that a patient will visit a health facility with tuberculosis diagnostic availability during their first visit. This metric is calculated by multiplying the share of care initiation at each health facility level and sector by the coverage of microscopy at that level and sector. The results at each level and sector are then summed to provide an overall metric for diagnostic availability at the point of care initiation (column 3, Figure 1).

The fourth column shows the share of facilities at each level and sector that provide tuberculosis treatment services (Figure 1). The list of facilities maintained by the NTP related to diagnostic coverage was also used for treatment coverage in step 4. As a result, diagnostic and treatment coverage were identical for the public sector. In addition to the NTP list of facilities with tuberculosis diagnostic and treatment coverage, the NTP also maintains a second data set that includes a list of private-sector general practitioners who provide tuberculosis treatment services. These data were included in step 4 to estimate treatment coverage in the private sector.

The fifth column calculates treatment service availability for each province (Figure 1). This step estimates the likelihood that a patient will initiate care in a facility that provides tuberculosis treatment services. This step is a multiplication of column 1 (care initiation) and column 4 (tuberculosis treatment coverage; Figure 1).

The sixth column shows the share of cases notified to the NTP among all estimated tuberculosis cases in Pakistan annually (Figure 1). Notified cases are reported by both public-sector and private-sector facilities, and the share reported by each sector is shown in this step. The remaining cases (in gray) are those that are missing or not notified to the NTP [7].

Finally, the seventh column shows the percentage of the estimated patients with tuberculosis who did (green) or did not (orange) successfully complete treatment (Figure 1). The remaining cases in (gray) are cases that are missing or not notified to the NTP [7].

### Limitations

The PPA attempts to approximate the steps a patient might take on the pathway to care, but there are several limitations to the analysis. The data on the number of facilities with tuberculosis services likely underestimate the number of facilities that actually provide diagnosis and treatment. The prevalence survey reported that at least one third of participants with tuberculosis symptoms received a diagnosis (based on smear or radiography findings) in the private sector, suggesting that diagnostic services may be more widely available than estimated by the PPA. Second, the PPA does not capture information on subsequent visits to health providers after care initiation or on the referral systems that are designed to facilitate follow-up visits. These secondary visits appear to be particularly important, owing to the misalignment between care initiation and the location of tuberculosis diagnostic and treatment services. Finally, care-seeking data were limited to only 4 of 5 provinces—Balochistan, Khyber Pakhtunkhwa, Punjab, and Sindh—and thus the PPA was only able to draw conclusions on the basis of the data available nationally and from those 4 provinces. Data from the NHA only provided information on general care-seeking patterns, which we used as a proxy for care seeking for tuberculosis symptoms. We assumed that general care-seeking patterns mirror tuberculosis care-seeking patterns in most cases (eg, for cough, fever, or mild pain). Further limitations of the PPA methods are described in more detail elsewhere [13].

## RESULTS

### Nearly 90% of Patients With Tuberculosis Initiated Care Seeking in the Private Sector

**Figure 2.**
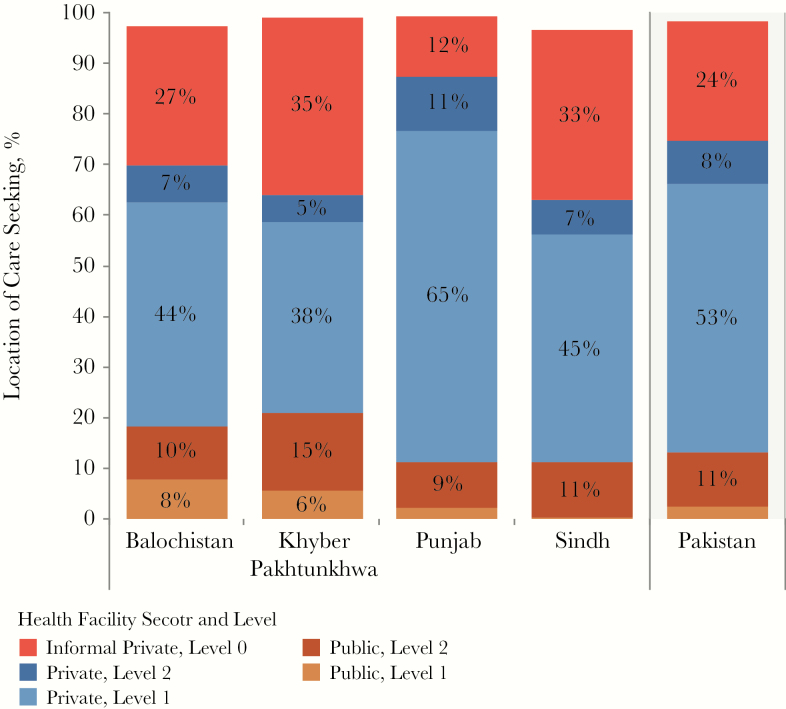
Care-seeking patterns, by province, for patients who visited a health facility during the 4 weeks before the health utilization survey conducted as part of the 2011–2012 National Health Accounts [14]. Responses from this survey were categorized using the standard health sector and level categorization described elsewhere in this article. Responses were available at the national level and for 4 provinces and were used in the patient-pathway analysis for each province (column 1, Figure 1). Percentages in columns might not sum to 100% because care-seeking data for facilities classified as “other” were excluded.

**Figure 3.**
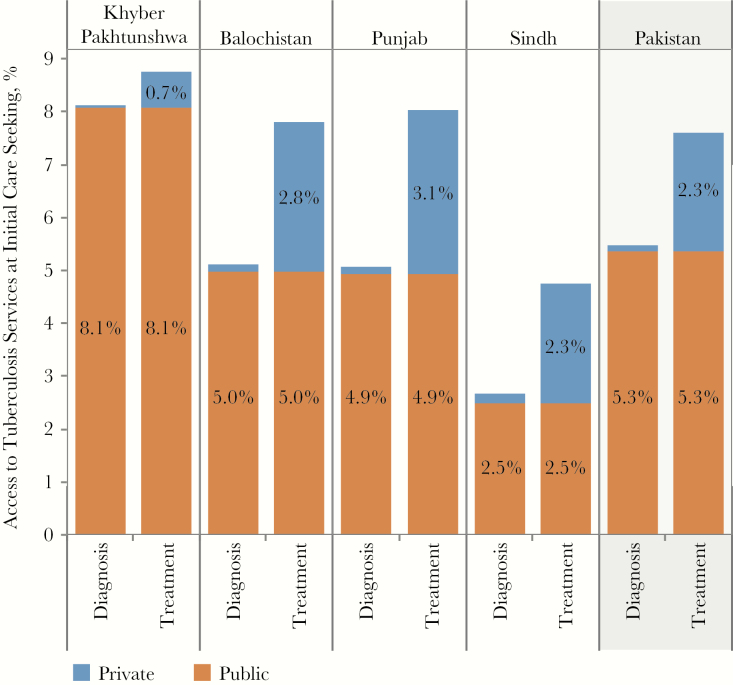
Access to diagnosis and treatment at initial care seeking, by province. The patient-pathway analysis was completed at the national level, as well as subnationally for 4 provinces. The figure shows the access to diagnosis and treatment at initial care-seeking metrics across each of these provinces (columns 3 and 5, Figure 1).

The most distinct finding from the PPA was the dominance of care initiation in the private sector. Of the 85% of patients who initiated care in the private sector, 61% went to formal providers, while 24% went to informal providers, such as pharmacists and traditional healers (column 1, Figure 1). In Balochistan, Khyber, and Sindh provinces, patients relied on the informal sector even more, with 27%, 35%, and 33%, respectively, initiating care in informal settings (Figures 2 and 3).

### Fewer Than Half of Public Sector Facilities Had Diagnostic Capacity

Although a smaller percentage of patients with tuberculosis initiated care in the public sector (13%) than in the private sector (87%), the limited access to diagnostic facilities within the public sector was noteworthy. Only 48% of public hospitals had microscopy services available. There was considerable subnational variation in the public sector with regard to the capacity for diagnosis. While 24% of L1 facilities (basic health units, basic management units, and dispensaries) had microscopy services in Punjab, this proportion was as low as 5% in Sindh. Similarly, only 23% of public hospitals in Sindh had microscopy services, compared with nearly 50% of hospitals in Punjab.

After aggregating the limited or undocumented capacity for diagnosis within the heavily utilized private sector and the partial coverage of microscopy in the public sector, we estimated that only 5.4% of patients accessed microscopy at the site of care initiation. In Khyber, where 21% of patients initiated care in the public sector, diagnostic access was slightly higher (8.1%) than the national average. In contrast, only 2.7% of patients accessed diagnostic services in Sindh upon care initiation, where 45% of patients initiated care in private clinics, with 33% initiating care in the informal private sector. Despite having the highest diagnostic coverage within public facilities, Punjab had the lowest level of public-sector utilization; only 11% of all care initiation visits were in public-sector facilities. In all provinces, facilities in the informal private sector offered no microscopy services, although there was a significant range across provinces in the utilization of informal private-sector facilities for care initiation, from 12% of initial visits in Punjab to 35% in Khyber Pakhtunkhwa.

### Treatment Availability Did Not Reflect Patient Care-Seeking Preferences

Nationally, 7.6% of patients initiated care in facilities that had the capacity to support tuberculosis treatment. This figure ranged from 4.8% in Sindh to 8.8% in Khyber Pakhtunkhwa, indicating a misalignment between the location of treatment services and patient preference for care facility, as proxied by the location of care initiation. Across provinces, treatment was available in the same public facilities that had diagnostic capacity, while private-sector facilities were more equipped to provide treatment than diagnostic services. While more than half of people (53%) initiated care in private clinics, only 2.8% of private clinics had tuberculosis treatment capacity. By comparison, 9.1% of private hospitals had tuberculosis treatment capacity, while only 8% of patients initiated care in those facilities. In 2 provinces, Khyber Pakhtunkhwa and Sindh, the availability of treatment services was much lower in the private sector, with <5% of private hospitals and diagnostic laboratories offering tuberculosis treatment, compared with 11% of private hospitals and diagnostic laboratories in Balochistan and 13% in Punjab. Only 7% of patients in Balochistan and 11% of patients in Punjab sought care in private hospitals and diagnostic laboratories. Nationally, tuberculosis treatment services were more widely available in the public sector; nearly 50% of public hospitals had treatment coverage. Nonetheless, very few patients sought care in the public sector.

## DISCUSSION

### Priority Must be Given to a Targeted Expansion of Private-Public Mix Programs to Engage Informal Providers and Lower-Level Private Clinicians in the Referral of Individuals With Presumptive Tuberculosis

The PPA not only confirmed the important role of the private sector in providing care in Pakistan, it also highlighted the extent to which patients utilize private clinicians and informal providers as entry points to care. Although lower-level private clinics and informal private providers account for two thirds of all initial care visits, they have limited capacity for tuberculosis diagnosis or treatment. However, without the engagement of private facilities in tuberculosis prevention, diagnosis, and care, they will continue to fail to detect and refer individuals presumed to have tuberculosis. There are already public-private mix models in Pakistan; this nascent collaboration between public and private facilities contributed approximately 22% of all notified tuberculosis cases in 2015 [17]. Formal assessments of their relative patient loads, cost-effectiveness, and treatment success have been reported in the literature [18]. The PPA provides further evidence to support the continued expansion of private-public mix programs and guides the targeting of future private-public mix program expansion toward models that incentivize referral of presumptive cases from informal providers to diagnostic sites, as well as the fortification of networks connecting private clinicians to nearby diagnostic facilities.

### Case Detection Rate Was High Given the Low Likelihood of Patients Reaching Tuberculosis Facilities With Diagnostic Capacity on Their First Visit

Despite the limited diagnostic availability at the locus of initial care, 63% of estimated tuberculosis cases in Pakistan were notified to the WHO in 2015. Data provided by the NTP suggested that <1% of private facilities had microscopy capacity. However, the prevalence survey found that 31% of patients with confirmed tuberculosis reported having had microscopy performed, and 43% reported undergoing radiography in the private sector [19]. The results suggest that the NTP database of microscopy sites may have missed numerous private-sector facilities with microscopy capacity. The prevalence survey found that, among patients with confirmed tuberculosis, 56% underwent radiography and 63% had a sputum smear performed at public-sector facilities [19]. This finding reflected higher rates of diagnostic confirmation from the public sector than from the private sector. Considering these findings together, it appears that patients made multiple visits to health facilities—possibly directed by active referral to sites with diagnostic capacity—to obtain a tuberculosis diagnosis.

There are several other possible explanations for the relatively high case notification rate, given the limited diagnostic infrastructure. These explanations include the active referral of patients, functional sample transport systems, and patient initiative to independently find a facility with tuberculosis diagnostic capacity. The first of these possible explanations hypothesizes the existence of an active yet informal referral network between facilities without and those with diagnostic capacity. The role of LHWs in identifying and referring individuals presumed to have tuberculosis from communities to qualified public providers has been well documented [4, 10–12]. Furthermore, a study that reviewed the tuberculosis diagnosis and treatment practices of private providers in Pakistan found that private health providers referred the majority (70.9%) of all presumptive tuberculosis cases for diagnosis [18]. Of the individuals with presumed tuberculosis referred from the private sector, 55% were referred for diagnosis to district NTP tuberculosis centers [17].

### A Case for Enhanced Tuberculosis Diagnostic and Treatment Capacity in the Public Sector

With both LHWs and the private sector referring presumptive cases to the public sector for diagnosis, a strong case emerges for further enhancing the availability of diagnostic technology within the public sector. When strong referral and sample transport mechanisms exist between health facilities where patients initiate care and those with diagnostic capacity, patients are likely to be detected and have treatment initiated in a timely manner. However, where these systems do not exist and patients need to find their own way, there could be significant delays in receiving proper diagnosis and care, leading to increased risk of transmission and poor treatment outcomes. This is particularly important to consider as the country moves to expand access to Xpert as an initial diagnostic and drug-resistance screening.

In 2015, between 32% and 74% of estimated tuberculosis cases were notified in their respective regions [9]. The variance in case notification rate is more extreme than the variance in capacity to diagnose or treat. A study on private provider practice found that only 29.1% of individuals with presumptive tuberculosis referred for diagnosis were treated by private providers [17]. It was not reported whether patients were referred to the public sector for treatment or were lost, although it is reasonable to assume that many transferred care to the public sector, given the relatively higher case notification rate from the public sector relative to the lower general utilization of the public sector.

Based on these findings, Pakistan should work to strengthen the capacity for both diagnosis and treatment in the public sector. A fortification of tuberculosis diagnostic and treatment capacity in the public sector will help resolve the paradoxical situation in which patients do not seek care in the public sector, because of perceptions of limited and poor quality public care, but are ultimately referred to public facilities, owing to the actual existence of higher-quality care in the public sector. Because so few people trust the public sector to provide quality care, it is also necessary to increase awareness of the public-sector capacity for tuberculosis diagnosis and treatment. Increased trust in the public sector should encourage more patients to initiate care in public facilities, reducing the number of patients lost between care initiation in the private sector and diagnosis and treatment in the public sector.

## Supplementary Data

Supplementary materials are available at *The Journal of Infectious Diseases* online. Consisting of data provided by the authors to benefit the reader, the posted materials are not copyedited and are the sole responsibility of the authors, so questions or comments should be addressed to the corresponding author.

## Supplementary Material

Supplementary Appendix

## Notes

***Supplement sponsorship.*** This work is part of a supplement sponsored by the Bill and Melinda Gates Foundation.

***Potential conflicts of interest.*** All authors: No reported conflicts of interest. All authors have submitted the ICMJE Form for Disclosure of Potential Conflicts of Interest. Conflicts that the editors consider relevant to the content of the manuscript have been disclosed.
