# Supplementary material for: Delivering Patient-Centered Care in a Fragile State: Using Patient-Pathway Analysis to Understand Tuberculosis-Related Care Seeking in Pakistan
Source: J Infect Dis. 2017 Nov 6;216(Suppl 7):S733–9. doi: 10.1093/infdis/jix380 (PMC5853661; doi:10.1093/infdis/jix380)
Supplement: Supplementary Appendix [file jix380_suppl_supplementary_appendix.docx]

| **Country: Pakistan** | | | | | |
| --- | --- | --- | --- | --- | --- |
| **Data Source** | **Survey Type** | **Survey Question** | **Reported Metric** | **Sample Size** | **PPA Step** |
| 2009-2010 National Health Accounts (private sector facilities) | The survey was conducted in four provinces of Pakistan. A sample of 2,160 primary sampling units (PSUs) was selected and 206,587 health care providers were estimated in Pakistan for FY 2009-10. | “Table 23 shows the estimated number and percentage of health care providers by three major categories and in case of hospitals by size respectively.” (p.46)   - Hospitals-big (>50 beds): 125 - Hospitals-small (<50 beds): 4255 - Out-Patient Service Providers: 196,843 - Lab & Diagnostic Service Providers: 5,489   Total: 206,712 | Private health providers 2009-10 by type, size and province  (Table 23) | N/A | Number of health facilities |
| 2011-2012 National Health Accounts | Two stage stratified random sampling scheme adopted to select n=8038 households, pertaining to 297 urban and 292 rural areas in all four provinces. There are 3,031 households reported, having no illness in recall period. | Public health facilities in Pakistan 2012   - Hospitals: 980 - Dispensaries: 5,039 - Basic Health Units: 5,449 - Rural Health Centres: 579 - MCH Centres: 851 - TB Clinics: 345 - Beds in hospitals & dispensaries etc.: 90,712   Population per bed: 1,665 | Number of public health facilities in Pakistan 2012  (Table 38) | N/A | Number of health facilities |
| 2011 -2012 National Health Accounts | Two stage stratified random sampling scheme adopted to select n=8038 households, pertaining to 297 urban and 292 rural areas in all four provinces. There are 3,031 households reported, having no illness in recall period. | Was a Health Care Facility accessed by any household member in the last 4 weeks? HE07: Type of Provider  Private sector provider   1. Private hospital 2. Private doctor clinic 3. LHV/nurse in private sec 4. LHW 5. Homeopath/Hakeem/Herbalist/Siana/Dai 6. Pharmacy/Shops 7. Laboratory   20. Other, Specify  Public sector provider   1. Government hospital 2. Dispensary/Maternal and Child Health Centre 3. BHW 4. RHC 5. THQ/DHQ 6. Tertiary, teaching or specialized hospital 7. Military Hospital 8. Social Security Hospital 9. Autonomous bodies/semi-govt. hospital 10. Don’t Know 11. Laboratory 12. Other, Specify   Annexure 15: Questionnaire of OOP survey 2011-12 (p.108) | Type of health care provider accessed in the last 4 weeks  (Table 27) | n=5007  Number of households that included a member who accessed a health care facility in the past 4 weeks  (8038 total households minus 3031 households that reported no illness during recall period=5007 households) | Step 1 –  Initial care seeking patterns |
| Pakistan NTP Lab Database | N/A | Internal dataset provided by NTP | TB Microscopy Services Coverage | - 1441 total TB health facilities providing microscopy and treatment services | Step 2 –  Coverage of microscopy among health facilities  +  Step 4 –  Coverage of TB treatment services |
| Internal NTP List of GPs with TB Treatment Services | N/A | Internal dataset provided by NTP | TB Treatment Services Coverage | - 1494 GP treatment centers - 2178 Functional GP treatment centers | Step 4 –  Coverage of TB treatment services |
| 2016 WHO Global TB Report | Annual report providing data on TB epidemiology, health systems and financing for 194 member state.  Notification location data available in table 4.2 of annual report.  Raw data accessed via Global TB Database available here:  <http://who.int/tb/data/en/> | N/A | Estimated burden – 510,000 cases  TB treatment coverage (case detection rate) – 63%  New and relapse notified cases –  323,856  Share of notified cases from private sector – 22%  Treatment success rate – 93% | | Step 7 –  Among estimated burden-notification source  +  Step 8 –  Among estimated burden-successfully treated cases |
